# Supplementary material for: Therapeutic timing limitations of postnatal darbepoetin in a valproic acid rat model of Autism Spectrum Disorder
Source: PLoS One. 2025 Nov 24;20(11):e0337294. doi: 10.1371/journal.pone.0337294 (PMC12643318; doi:10.1371/journal.pone.0337294)
Supplement: Attachment — Submitted filename: Response to Reviewers.docx [file pone.0337294.s006.docx]

**Dear Dr. Sirén,**

Thank you for the opportunity to revise our manuscript, **"Therapeutic Timing Limitations of Postnatal Darbepoetin in a Valproic Acid Rat Model of Autism Spectrum Disorder"**. We are grateful for the insightful feedback from you and the reviewers, which has substantially improved the scientific rigor and clarity of our work. We have carefully addressed each point raised and made additional minor edits throughout for clarity. All modifications are marked using "Track Changes" in the accompanying document.

Please find below our point-by-point response to the comments.

**Response to Journal Requirements**

**Funding statement:** As requested, we have removed the funding statement from Acknowledgments. As previously disclosed, *“Our study is funded by Erciyes University Scientific Research Projects Coordination Unit”*. To elaborate as requested we’d like amend the statement as **“Our study is funded by Erciyes University Scientific Research Projects Coordination Unit. The funders had no role in study design, data collection and analysis, decision to publish, or preparation of the manuscript.”**

**Data availability:** We are pleased to confirm that all raw data and statistical outputs supporting our findings have been deposited in a public Zenodo repository (DOI: 10.5281/zenodo.15727029). The data are currently under embargo but will be made fully and publicly available without restriction upon acceptance of the manuscript, ensuring compliance with PLOS ONE's open data policy. Should the Editor require access to the data during the review process, we would be happy to provide immediate access via Zenodo's sharing function.

**PLOS ONE Style Requirements:** We have reviewed the manuscript to ensure it fully complies with the journal's style and formatting guidelines.

**Figure Captions:** We have ensured that each figure in the manuscript is accompanied by a separate and complete caption.

**Response to Academic Editor**

***Point 1: “Please elaborate on your choice to use darbepoetin instead of EPO for your studies, especially since because of its pharmacokinetics darbepoietin is known to induce a robust increase in red cell production and hemoglobin. Also describe and discuss more detailed the reasons for the selection of dose of darbepoietin and the time window of treatment as well as the potential consequences of these decisions for the study outcome.”***

**Response:**

- **Darbepoetin selection and dose:** We have added substantial detail to the Introduction (page 4-5, paragraph beginning ***"We selected darbepoetin alfa..."***) clarifying darbepoetin's (DPO) neuroprotective properties, extended half-life, clinical availability, and dose calculation based on established bioequivalence ratios and prior neuroprotective protocols.
- **Treatment window:** We have expanded the Introduction (page 4, paragraph beginning ***"A major challenge..."***) with detailed, literature-backed justification for selecting the P21-25 window, which corresponds to human post-diagnostic period and key neurodevelopmental milestones.
- **Outcome:** We have added comprehensive discussion in two sections on page 22 ***"Hematological response"*** and page 23 ***"Therapeutic implications"***, contextualizing our findings within existing literature and discussing non-erythropoietic alternatives. Following Reviewer #2's valuable comment, we also extracted hematocrit data from the hemoglobin reader (Mission Ultra Hb, ACON Laboratories, Inc., San Diego, CA, USA). In line with existing literature where hematocrit is the primary hematological parameter, we shifted our focus to hematocrit values in the Discussion and main figures. Hemoglobin measurements remain summarized in Results and fully detailed in Supporting Information S4 File alongside Hematocrit data.

***Point 2: “Please explain in more detail the pharmacological differences between EPO and darbepoetin. Please also discuss more in detail the safety profile of EPO in comparison to its analogues.”***

**Response:**

- **Pharmacology:** The Introduction (page 4-5, paragraph beginning ***"We selected darbepoetin alfa..."***) details DPO’s additional sialic acid residues, longer half-life, clinical dosing and varying applications for neuroprotective use.
- **Safety profile:** The ***"Hematological response"*** section (Discussion on page 22), includes a detailed comparison of hematological effects across EPO/DPO studies and discusses our outcome. On page 23 under ***"Therapeutic implications"***, we further detail the discussion with human studies and the experimental non-erythropoietic analogues as translational options.

**Point 3:** ***“Please also elaborate more in detail in the introduction the advantages and disadvantages of your animal model for autism spectrum disorders (ASD).”***

**Response:** We have added substantial detail for the VPA model in the Introduction (page 3, paragraph beginning ***"The prenatal VPA model..."***), elaborating on our model’s advantages and limitations. We further acknowledged the VPA model’s and the male-only cohort’s limitation in the Discussion, on page 24, under ***“Limitations”***.

**Point 4: *“The details of the behavioral paradigms should be more elaborated in the methods section.  Also please describe the relevance of the behavioral tests for your disease model.”***

**Response:** In the Introduction, page 5, paragraph beginning with ***“To characterize…”***, we expanded the description of the behavioral paradigms and clarified their relevance to ASD-related domains, with supporting citations to the relevant literature.

We have added more detail for the behavioral paradigms in the methods section. We have renamed ambiguous behavioral parameters to use more standard and descriptive terminology. During revision, we identified an error in the reported dimensions of the three-chamber apparatus. The manuscript previously stated 60 × 30 × 30 cm, which were dimensions from earlier pilot work in our laboratory. We have corrected this to the actual apparatus used in this study measured 120 × 60 × 35 cm. This correction does not affect any data or analyses, as all EthoVision XT tracking and zone calibrations were performed using the actual apparatus dimensions, and all behavioral measurements remain unchanged.

**Point 5: *“Discussion on sex-specific effects is not clearly justified if only male mice were tested.”*
Response:** We have removed the introductory information about sex-dependent prevalence from the Introduction, and have ensured we do not discuss sex-specific effects in the Results or Discussion.

**Point 6: *“Pay attention to the correct labelling of all axes and legends in the figures.”***

**Response:** We have replaced all figures with versions that comply with PLOS ONE guidelines, ensuring: All axes are properly labeled with units, Legends are complete and clear, Figure quality meets journal standards, Statistical indicators are appropriately marked and All figures (Figs 1-8) have been updated accordingly.

**Point 7: *“Please add all post hoc comparisons following the two-way ANOVAs, perhaps in a table or a supplementary file.”***

**Response:** We have prepared comprehensive supplementary excel files containing all statistical analyses for: Three- Chamber Test (S1 file), Morris Water Maze Progression (S2 file), Morris Water Maze Day-based (S3 file) and Hematological parameters (S4 file).

**Response to Reviewer #1:**

We are grateful to the Reviewer for the time, effort, and insightful feedback provided. We have found the comments to be exceptionally constructive, and they have helped us to substantially improve the scientific rigor, clarity, and overall impact of our work.

***Main comment: “The authors present a first-of-its kind study of darbapoetin alfa, an erythropoietin derivative, on autism-related behavior phenotypes in the valproic acid models in a development context. The authors are driven by the observation that erythropoietin and its derivatives seem to be able to correct cellular dysfunctions that appear to play a role in autism and wish to explore this further. The authors correctly point out that previous studies used models with important limitations or did not look at the chronic importance of erythropoietin and its derivatives at a developmentally relevant time point, and the experiments seem to be well-conducted and well-analyzed, but this study also has key important limitations that are under-discussed and affect the overall impact of the paper. The authors find that at a particular dosage of darbapoetin for five days starting on PD21 has very slight impacts on social behaviors and minor impacts on performance in the Morris water maze. These results suggest that this particular dosage scheme has no chronic impacts on the narrow range of ASD-related behaviors tested. The authors explain these null results by suggesting that appropriate developmental time period has passed (though they buffer this remark by pointing out that other erythropoietin drug experiments don't suffer from this problem), that there were effects by increased hematopoietic load, or that that are vague issues regarding baseline sociability or receptor targeted. However, there may be a simpler explanation: the dosage is simply not large enough or has not been delivered for long to have the effects that they wish to rescue, but that the dosage is already too high to be safely administered and it would be unsafe to increase the dose to test this hypothesis. This explanation is not clearly explored in the paper, and any consideration of this finding should lay this out more clearly while also admitting the extremely limited nature of the finding.”***

**Comment 1: *“In the last sentence of the abstract, the authors suggest that the therapeutic window for EPO analogues may close earlier, but for the reasons I lay out above, and using evidence they lay out in the discussion, this statement is not supported by the rest of the paper. This should be changed.”***

**Response:**  We have improved our discussion with this valuable comment (Below, at response to comment 2), and have revised the Abstract and Conclusions to reflect both our primary interpretation and this acknowledged uncertainty.

**Comment 2: *“In the abstract, and at other locations in the paper, the authors raise the point that EPO analogues may be unsafe for a juveniles. This is not a novel observation--there is a lot of literature about the safety issues surrounding EPO at dosages required for neuopsychiatric differences, and these findings have driven the search for safer alternatives that yield similar results. The authors should do a more careful review of this literature and use this to provide context for their results.”***

**Response:** We have significantly revised our discussion in the previously mentioned two sections: ***"Hematological response"*** (page 22): We have provided detailed comparisons showing that our dose and hematocrit response are comparable to studies reporting neuroprotection at the high end of published ranges and achieved behavioral improvements using an equivalent cumulative dose administered at the earlier P1-5 window. We have toned down our initial characterization of the hematological response as inherently unsafe and we refrained from presenting it like a novel observation and interpreted that treatment timing is the primary constraining parameter.

***"Therapeutic implications"*** (page 22): We also acknowledged that while timing appears to be the critical factor, we cannot definitively exclude the possibility that higher doses or longer treatment duration might compensate for delayed intervention, because the pronounced hematological response constrains further dose escalation on safety grounds. We discussed non-erythropoietic analogues as a potential avenue for future research to address the limitation in this context. We cited Juul et al.'s clinical trial showing a measurable increase in thrombotic events in neonates, still emphasizing the need for caution when translating these therapies.

**Comment 3: *“In the introduction, the authors spend time talking about sex differences despite the fact that only males are used in the their paper. They should remove these statements.”***

**Response:** We have removed the information about sex-dependent prevalence from the Introduction.

**Comment 4: *“In the introduction, the authors should point out the limitations of using VPA to represent the broader autism patient population.”***

**Response:** We adressed this limitation in the Introduction (page 3, paragraph beginning "***The prenatal VPA model..."***). We further acknowledged the VPA model’s and the male-only cohort’s limitation in the Discussion, on page 24, under ***“Limitations”***.

**Comment 5: *“In the introduction, the authors should spend slightly more time talking about the differences between DPO and EPO.”***

**Response:** The Introduction (page 4-5, paragraph beginning ***"We selected darbepoetin alfa..."***) now details DPO’s additional sialic acid residues, longer half-life, clinical dosing and varying applications for neuroprotective use with citations from previous DPO and EPO studies.

**Comment 6: *“How dose the dosage actually compare to the two studies referenced in the introduction? Because a key limitation to this study is the choice of the dosage itself, the authors should spend more time talking about their choices with specific references to studies where DPO has made a difference neurobehaviorally.”***

**Response:** We have substantially strengthened our dose justification with specific comparisons in The Introduction (page 4-5, paragraph beginning ***"We selected darbepoetin alfa..."***) with bioequivalence ratio aligned with previous neurodevelopmental study, a specific reference to a study where Cherian et al. tested DPO across 2.5–50 µg/kg in adult rats, reported robust histological and neurological recovery with 25 and 50 µg/kg similarly. We further discussed the dose-efficacy details under ***"Hematological response"*** (Discussion, page 22).

**Comment 7: *“In the methods section, there is a suggestion that testing spanned three consecutive days, but there are ony two experimental days mentioned. Please correct this.”***

**Response:** We have corrected this error. The Methods section now accurately states "Testing spanned two consecutive days”.

**Comment 8: *“In the results, there is a reference to experiments (a "subsequent novelty trial" in the three-chamber that are not described in the methods. These experiments should be described there, and their findings should be included in the manuscript (perhaps in a supplemental figure). The way these findings are discussed in the methods is not appropriate and they should be described completely. The authors should also talk about why they decided to describe variability.”***

**Response:**

**Novelty test:** We have addressed this important gap by adding a complete description of the Social Novelty Phase in page 9. We reported its findings in the supplement file S1, under “Novelty” sheet and revised the paragraph in Results beginning ***“A subsequent social novelty…”*** on page 15.

**Variability:** We have added under ***“Behavioral findings”*** (Discussion, page 21), paragraph beginning with ***“In the three-chamber test”*** an explanation for why we decided to describe this variablity and added a brief discussion about this effect.

**Comment 9: *“In the results, the authors use unusual terminology to describe the social and non-social objects in phase 2 of the experiment. Rather than "intruder" and "familiar" they should use other language.”***

**Response:** We have systematically replaced this terminology throughout the manuscript and figures:

"Intruder" → "Social" (or "Stranger 1" in Methods when referring to the specific conspecific)

"Familiar" → "Non-social" (or "empty cage" in specific contexts)

**Comment 10: *“In the results, the authors spend a bit of time speculating as to why the Morris water maze results turned out the way it did. This should be moved to discussion.”***

**Response:** We have summarized and moved this discussion from the Results section to the Discussion, under ***“Behavioral findings”*,** page 22, paragraph beginning ***"A nuanced aspect..."***.

**Comment 11: *“The day-specific comparisons should either be described in their entirety or removed.”***

**Response:** We have removed the summary paragraph describing day-specific comparisons from the Results section. We retained the Day 5 probe trial results as these represent a distinct experimental phase. We added a clear statement in Results (page 18-19, end of ***“Reversal Learning”*** section) about this decision, and have moved this data to the supplementary file S3.

**Comment 12: *“In the discussion, the authors should thoroughly address the critiques I outline at the beginning of this critique.”***

**Response:** We have significantly revised our discussion in two sections as previously described under Response to Comment 2. We have added the ***"Hematological response"*** ***"Therapeutic implications"*** sections to Discussion, page 22, adressing thesepoints. We have also revised our Abstract and Conclusion sections accordingly.

**Comment 13: *“In the discussion, the authors should address how these findings compare to other neurobehavioral findings with DPO.”***

**Response:** In the Introduction, page 4-5, paragraph beginning ***"We selected darbepoetin alfa..."***, we have cited other DPO neuroprotection studies and emphasized that to our knowledge, this is the first study to evaluate DPO in a neurodevelopmental model. In the Discussion, page 22, under ***"Hematological response"***, we detailed Cherian et al.’s reported neurological recovery with 25-50 µg/kg DPO doses and discussed our different outcomes. Under the same section, we emphasized that the previous studies primarily simulate acute neuroinflammation and function more as neurotoxicity paradigms, making neurological outcomes hard to compare.

**Comment 14: *“In all of the figures, the y-axes should be labeled appropriated throughout.”***

**Response:** We have labeled y-axes in all figures with appropiate names and units.

**Comment 15: *“For clarity, it would be useful for the authors to report all post hoc comparisons following the two-way ANOVAs, perhaps in a table or a supplementary file.”***

**Response:** We have prepared comprehensive supplementary excel files containing all statistical analyses for: Three- Chamber Test (S1 file), Morris Water Maze Progression (S2 file), Morris Water Maze Day-based (S3 file) and Hematological parameters (S4 file).

**Response to Reviewer #2:**

We are grateful to the Reviewer for the time, effort, and insightful feedback provided. We have found the comments to be exceptionally constructive, and they have helped us to substantially improve the scientific rigor, clarity, and overall impact of our work.

**Comment 1: *“Model validity and scope of inference***

***The prenatal VPA rat is a well-established, widely used environmental/teratogenic ASD-relevant model. Please specify gestational day(s), dose, and strain, and discuss how these parameters align with canonical protocols, as outcomes are highly timing- and dose-sensitive.***

***Because ASD is heterogeneous, the manuscript should avoid general conclusions about “ASD” per se. Consider framing results as domain-specific within this model. If feasible, discuss (or acknowledge as a limitation) how findings might compare with complementary models (genetic or immune-activation), which could strengthen mechanistic inference.”***

**Response:**

We have added substantial detail for the VPA model in the Introduction (page 3, paragraph beginning ***"The prenatal VPA model..."***) about the dose, timing and strain details with canonical protocols cited. In the same paragraph, we noted that the VPA does not represent the broader heterogeneity of ASD. We further acknowledged and discussed the VPA model’s and the male-only cohort’s limitation in the Discussion, on page 24, under ***“Limitations”***.

**Comment 2: *“Rationale for DP regimen and pharmacology”***

a) ***Provide a stronger justification for the dose, route, and timing (PND21–25). Is this window expected to influence circuits implicated by prenatal VPA exposure?***
b) ***Include, if available, pharmacokinetic or pharmacodynamic markers (e.g., hematocrit/reticulocytes, EPO-receptor pathway readouts, brain exposure) to confirm target engagement.***
c) ***Discuss literature on earlier neonatal or perinatal EPO-family interventions and whether an earlier window might be necessary.***
d) ***Clarify whether sex-specific effects were considered; if both sexes were studied, analyze sex as a factor or justify pooling.***

**Response:
a) Timing and Pharmacology:** In the introduction, page 4, paragraph beginning ***“A major challenge…”***, we detailed the rationale on PND21-25; it’s alignment with human diagnosis age with referenced studies, and the dynamic neural events in this period like synaptic pruning, which is shown to be distrupted in ASD and that can be modulated by EPO receptor signaling. In the next paragraph, page 4-5, beginning ***"We selected darbepoetin alfa..."***, we detailed pharmacological details of DPO, detailed our dosing regimen and how it aligns with similar studies. We further made comparisons with previous studies on page 22, under ***“Hematological response”***, discussing that the prominent hematological reponse is an expected part of high neuroprotective doses and compatible with neuroprotective efficacy when intervention timing is optimal.

**b)** **Hematocrit/markers:** Following this valuable comment, we also extracted hematocrit data from the hemoglobin reader. In line with existing literature where hematocrit is the primary hematological parameter, we shifted our focus to hematocrit values in the Discussion and main figures. Hemoglobin measurements remain summarized in Results and fully detailed in Supporting Information S4 File alongside Hematocrit data. Unfortunately we were not able to measure other markers.

**c) Earlier windows:** In the introduction, page 3-4, paragraph beginning ***“Erythropoietin (EPO)…”***, we cited the Haratizadeh et al.’s EPO study on neonatal VPA rats which improved behavioral outcomes. In the next paragraph beginning ***“A major challenge…”***, we explained that ASD can only be diagnosed around 2-3 years of age, by which time many sensitive windows have passed, as a result, the neonatal treatment protocols may not translate realistically for clinical applications.

**d) Sex factor:** In the Introduction (page 3, paragraph beginning ***"The prenatal VPA model..."***), we have clarified our male-cohort choice. We later acknowledged the VPA model’s and the male-only cohort’s limitation in the Discussion, on page 24, under ***“Limitations”***.

**Comment 3: *“Behavioral battery adequacy for ASD-relevant domains. The combination of three-chamber sociability and MWM addresses social approach and spatial learning but does not cover other core ASD-relevant dimensions. Please consider (or acknowledge as limitations) assays of: Restricted/repetitive behavior (e.g., self-grooming scoring, marble burying, stereotypy quantification), Communication (e.g., juvenile/adult ultrasonic vocalizations), Sensory processing and sensorimotor gating (e.g., acoustic startle/PPI)”***

**Response:**

In the Introduction on page 5, paragraph beginning with ***“To characterize…”*** we provided rationale for the behavioral assays we selected for this study. We acknowledged the limited scope of our behavioral assays in the Discussion, on page 24, under ***“Limitations”***.

While we did not include dedicated stereotypy assays, we extracted “Alternation rate” parameter, which is commonly used in Y-maze paradigm to asses spatial working memory and exploratory behavior, adapting it to the three chamber test as we detailed in the Methods section (page 9-10) and we discussed it under ***“Behavioral findings”*** on page 21 as: *“Additionally, VPA-exposed animals showed reduced alternation rates and increased immobility in two-way comparisons, which can be interpreted as proxies for restricted and repetitive behavior domains in ASD. DPO treatment did not significantly modify these secondary behavioral measures.”*

We believe these revisions have substantially strengthened the manuscript's scientific rigor, interpretive framework, and transparency. We are grateful to the Editor and the Reviewers for the opportunity to improve our work through this revision process.

Sincerely,

Ömer Yusuf İPEK and co-authors
